# Development of 6 DOF Displacement Sensor Using RUS Parallel Mechanism

**DOI:** 10.3390/s21113832

**Published:** 2021-06-01

**Authors:** Donghyun Kim, Sunghyun Choi, Dongwon Yun

**Affiliations:** Department of Robotics, Daegu Gyeongbuk Institute of Science and Technology (DGIST), Daegu 42988, Korea; kdhhouse@dgist.ac.kr (D.K.); csh7369@dgist.ac.kr (S.C.)

**Keywords:** RCC (Remote Center Compliance), 6DOF distance sensor, peg-in-hole, Stewart System, singularity, RUS system

## Abstract

Nowadays, many types of manipulators have been developed and used in lots of production processes. Force-based control methods or additional mechanical devices called Remote Center Compliance (RCC) have increased the system’s compliance and accuracy. However, the force-based control method’s operating speed is low, and the RCC cannot measure deflection. Thus it cannot calculate the position of the end-effector accurately. For accurate force and position control, it is necessary to measure the deflection of the RCC and to perform this, a different type of device than the existing RCC is required. This paper presents the necessity and possibility of developing an RCC capable of measuring the displacement of the end-effector and showing the displacement sensor’s feasibility using a 6 DOF parallel mechanism. In particular, we suggest that it is possible to make devices cheaper and more compact by using angular displacement sensors. Finally, we show the possibility of use in actual industrial sites through peg-in-hole simulation using the device.

## 1. Introduction

Over the last few decades, manipulators have been developed and used in many production processes. Recently, robots that can collaborate with humans have been researched. The robot cannot participate in every part of the process because of the limitations of the robot’s flexibility. In particular, it is easy to fail the assembly process despite minimal errors, which can cause serious damage to the robot and assembly parts. It is essential to use force control techniques when measuring the reaction forces generated during assembly and control [1,2]. Additionally, hybrid control techniques are used when combining position control and force control [3]. These techniques have the advantage of having good accuracy. However, it needs a long time to conduct the controls. In addition, six-axis force and torque sensors attached to the end-effector are costly.

Another approach is using remote center compliance (RCC) that provides flexibility to the end-effector through the mechanical structure [4,5]. RCC has no electronic input and output and has a system composed of compliant materials in parallel. RCC absorbs the reaction force generated during the assembly process through the deformation of the spring structure. Thus, it is possible to perform the assembly task while preventing the disrepair of robots and assembly parts. In addition to the assembly process, RCC is essential when performing picking motion to protect the gripper from external forces. Research on grippers that pick up atypical objects, including soft grippers, has been studied in recent years [6]. These grippers are more easily exposed to reaction forces, so the RCC is essential for them. However, due to compliance, the end-effector changes and cannot measure how much it is. That means we cannot know the position and orientation of the end-effector exactly during tasks.

The 6 DOF parallel mechanism has been steadily researched and developed since Stewart designed it [7]. The platform has been developed in various types with different joint combinations, with the kinematics, singularity, statics, and workspace studied for each mechanism [8,9,10,11]. Until these studies were sufficiently advanced, parallel mechanisms were difficult to use as sensors. Especially, the forward kinematics of parallel mechanisms have been hard to solve because of their nonlinear simultaneous equations. Besides, singularities appear differently depending on the geometry shapes and the joint configuration, which takes a long time to analyze. At present, many studies have been conducted so that various forward kinematics solutions of parallel mechanisms exist using neural networks [12], vector regression [13], and high order polynomials [14]. Additionally, when designing parallel mechanisms, singularities can be minimized according to the geometric shapes [15]. The advancement of computers has significantly reduced computation time, enabling parallel mechanisms to be used as sensors. 

**Figure 1:**
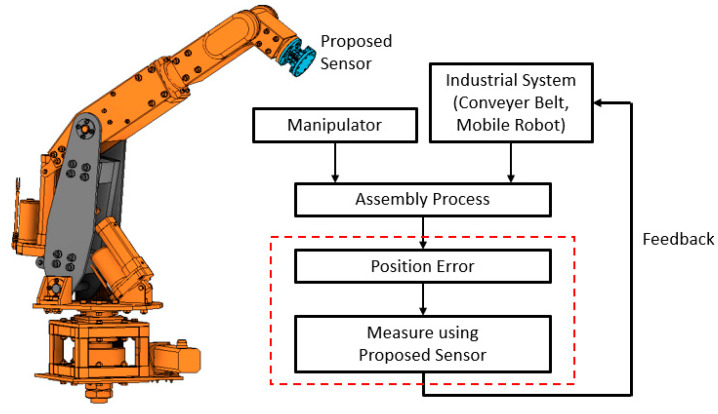
Concept of self-teaching system.

This paper deals with RCC development that can measure displacement using a 6 DOF parallel mechanism. Lee and Nayak developed an SPS (Spherical Prismatic Spherical) type parallel mechanism measuring displacement using linear displacement sensors [16,17]. We suggest that it is possible to develop an inexpensive and compact displacement sensor by using potentiometers as angular displacement sensors. It can be used to automate teaching robots in the assembly process by sensing position errors and feeding them back to the system, as shown in Figure 1.

The contents of the paper are organized as follows. Section 2 analyzes the kinematics of the RUS (Revolute-Universal-Spherical) parallel mechanism using the transformation matrix and proposes that it is possible to get the position and orientation of the end-effector from the six potentiometers. Additionally, we show the singularities found in this process and how to avoid them. Section 3 describes the proposed sensor design, the experimental procedure to validate it, potential verification through the use of the peg-in-hole system, results, and discussion of them. Section 4 will cover the conclusion and future work.

## 2. Methods

The principle of the proposed sensor is based on the kinematics of a 6 DOF parallel mechanism. When using a 6 DOF parallel mechanism as an actuator, inverse kinematics is used to produce the desired motion. On the other hand, when used as a sensor, it is required to solve forward kinematics to obtain six positions and orientations accurately. We solved the inverse kinematics of the parallel mechanism analytically and solved the forward kinematics using the numerical method. Then the result of the forward kinematics was verified by comparing it with the inverse kinematics. Additonally, the singularity must be removed to get one output from one input. We eliminated singularity by changing the direction of the revolute joint and verified it experimentally. 

### 2.1. Inverse Kinematics

**Figure 2:**
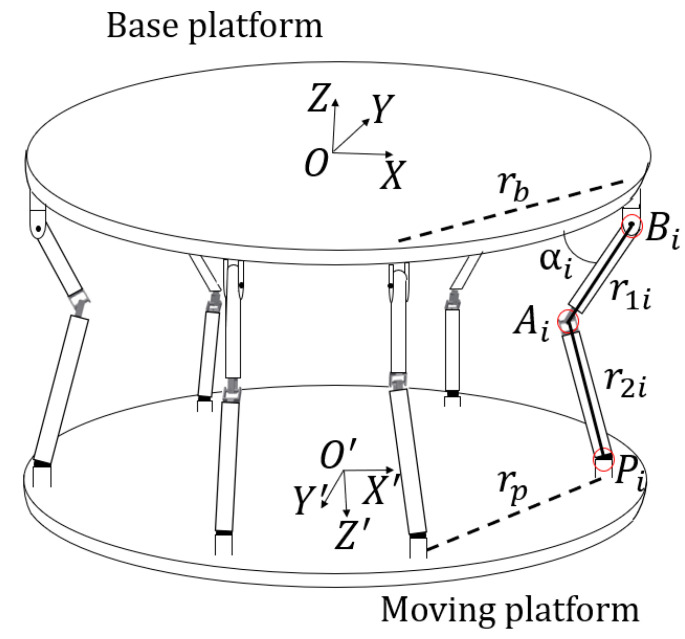
Schematic diagram of Revolute-Universal-Spherical (RUS) parallel mechanism.

Figure 2 shows the schematic diagram of the 6-6 RUS parallel mechanism. The O−XYZ coordinate system is a fixed coordinate system attached to the base platform. The $O^{\prime }-X^{\prime }Y^{\prime }Z^{\prime }$ coordinate system is a moving coordinate system attached to the moving platform. $B_{i},\;P_{i}$ represent the vertices of the base platform and moving platform respectively and $A_{i}$ is defined as the point where two links $r_{1i}$ and $r_{2i}$ contact. $B_{i},\;A_{i}$ and $P_{i}$ are composed of revolute, universal and spherical joints respectively, forming a parallel mechanism. Let the position of the point O′ with respect to the O−XYZ coordinate system be ${\left[x\;y\;z\right]}^{T}$, with orientation angles of ${\left[\psi \;\theta \;\varphi \right]}^{T}$. Then the transformation matrix from the O−XYZ coordinate system to the $O^{\prime }-X^{\prime }Y^{\prime }Z^{\prime }$ coordinate system is represented by Equation (1).

(1) $$T=\left[\begin{array}{cccc} q_{11} & q_{12} & q_{13} & x\\ q_{21} & q_{22} & q_{23} & y\\ q_{31} & q_{32} & q_{33} & z\\ 0 & 0 & 0 & 1 \end{array}\right]$$

(2) $$\begin{array}{l} q_{11}=c\theta c\varphi \\ q_{12}=-c\theta s\varphi \\ q_{13}=s\theta \\ q_{21}=c\psi s\varphi +s\psi s\theta c\varphi \\ q_{22}=c\psi c\varphi -s\psi s\theta s\varphi \\ q_{23}=-s\psi c\theta \\ q_{31}=s\psi s\varphi -c\psi s\theta c\varphi \\ q_{32}=s\psi c\varphi +c\psi s\theta s\varphi \\ q_{33}=c\psi c\theta \end{array}$$

**Figure 3:**
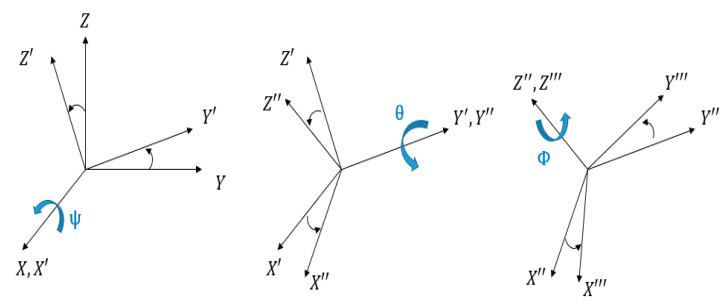
Representation of the *X*–*Y*–*Z* Euler Angle.

In Equation (2), the variables $q_{ij}$ for i,j=1, 2, 3 are elements of the product of the rotation matrix along X−Y−Z axis as shown in Figure 3 The length of the base platform’s one side and moving platform are $r_{b}$, $r_{p}$ respectively and the revolute joint angles are $\alpha_{i}$. Then the positions of $A_{i},\;P_{i}$ with respect to O−XYZ are given as Equations (3) and (4). Equation (5) shows the terms in Equations (3) and (4)

(3) $${\;}^{O}A_{i}={\;}^{O}B_{i}+\left[\begin{array}{c} r_{1i}cos\alpha_{i}\cos\left(\frac{\pi }{3}\left(i-1\right)\right)\\ r_{1i}cos\alpha_{i}\sin\left(\frac{\pi }{3}\left(i-1\right)\right)\\ -r_{1i}sin\alpha_{i}\\ 1 \end{array}\right]$$

(4) P Oi=T P O′i

(5) B Oi=[rbcos(π3(i−1))rbsin(π3(i−1))01] ,P O′i=[rpcos(−π3(i−1))rpsin(−π3(i−1))01]

Finally, we could get Equation (6) which represents the distance between two points $A_{i}$ and $P_{i}$. Simplifying Equation (6), we could get six independent equations which form Equation (7). Equation (7) often appears in kinematics and the solution is well known as the tangent half-angle substitution. If we define $t_{i}=tan\frac{\alpha_{i}}{2}$, the quadratic equations with respect to $t_{i}$ are found as Equation (8).

(6) ‖A Oi−P Oi‖=r2i

(7) $$E_{i}=F_{i}cos\alpha_{i}+G_{i}sin\alpha_{i\;}$$

(8) $$E_{i}=F_{i}\left(\frac{1-t_{i}^{2}}{1+t_{i}^{2}}\right)+G_{i}\left(\frac{2t_{i}}{1+t_{i}^{2}}\right)$$

Equation (8) could be easily solved from the quadratic formula and analytical solutions for α could be obtained by substituting $\alpha_{i}=2tan^{-1}\left(t_{i}\right)$ again. From the quadratic formula, $\alpha_{i}$ had two solutions. One was the shape of which the two links $r_{1i}$ and $r_{2i}$ bend inward and the other one was opposite so that the two links $r_{1i}$ and $r_{2i}$ extended outward.

### 2.2. Forward Kinematics

In general, the forward kinematics of parallel mechanisms are hard to solve since they contain nonlinear simultaneous equations. Therefore, a numerical method was used to solve the nonlinear equation. We also solved the forward kinematics of the RUS parallel mechanism using the numerical method with MATLAB. We could use this method because computers are fast enough to handle the computations in real-time. Besides, in the numerical method, it is possible to derive different solutions depending on the initial value. We did not need to consider this problem because the fabricated sensor measured a small displacement within 10 mm. In this study, the numerical solutions were obtained by using the fsolve function of MATLAB based on the Levenberg-Marquardt algorithm. In this process, six nonlinear simultaneous equations involving position and orientation variables were given as Equation (9).

(9) fi(x, y, z, ψ, θ,φ)=‖A Oi−P Oi‖−r2i=0

Equation (9) represents the distance relationship between two points. To evaluate the numerical solution, the revolute joint angles obtained from the inverse kinematics on the arbitrary setting position and orientation were put back into the forward kinematics as inputs. Then, we solved the forward kinematics and checked whether they were correct. Table 1 shows the results. The units of position [x y z] are millimeters and those of orientation [ψ θ φ] are degrees. Results are equal within the error range.

## 3. Design and Experiment

### 3.1. Avoiding Singularities

**Figure 4:**
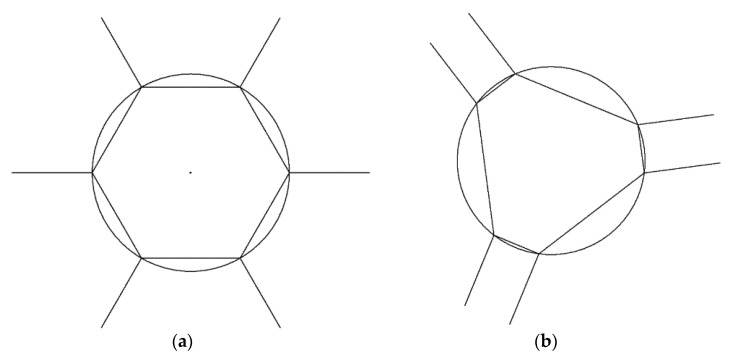
Top view of the prototype model (**a**) and modified model (**b**).

Figure 4a shows the top view of the prototype model, and Figure 4b shows the modified model respectively. The dotted line shows the directions of the revolute joint. Jiang defined Figure 4a as a regular type and mathematically showed architecture singularities in the SPS parallel mechanism. We found that the same singularities appear in the RUS parallel mechanism, i.e., the moving platform could twist like a screw motion along the *z*-axis when the base platform and the moving platform were parallel [18]. We modified the base platform configuration (see Figure 4b) to a semi-regular shape to solve this problem.

In the case of parallel platforms, an asymmetric shape is recommended to avoid singularity. Jiang mathematically found that the regular hexagonal shape shown in Figure 4a had singularities in many sections due to the symmetry [15]. The singularity problem could be reduced using asymmetric shapes. However, if the values such as the length and angle of each link were changed a lot, problems such as the difficulty of assembly and instability of the initial position may occur. The semi-regular type proposed in this paper could solve the singularity. It could avoid the difficulty of assembly and maintain the initial position since it did not change the shape significantly.

Table 2 shows the difference between the model when the singularity problem was solved or not. From this result, we could see that the singularity problem in the prototype model did not occur in the modified model. In an ideal case where singularity does not occur, 6 potentiometer values and the x, y, z, roll, pitch, and yaw values should have a 1:1 correspondence. In the prototype model, the shape was designed based on a regular hexagon. Thus, the singularity occurs, and the same angle sensor value could induce various results. Ideally, the result of the prototype model value in Table 2 would be (0 0 −25 180 0 0). However, a different solution was obtained when the initial value of the solver was different. The reason was that the *z*-axis and yaw-axis are coupled at the corresponding position. In order to prevent such singularity, it was necessary to use an asymmetric model. The singularity problem did not occur with the modified model.

### 3.2. Design of Displacement Sensor

**Figure 5:**
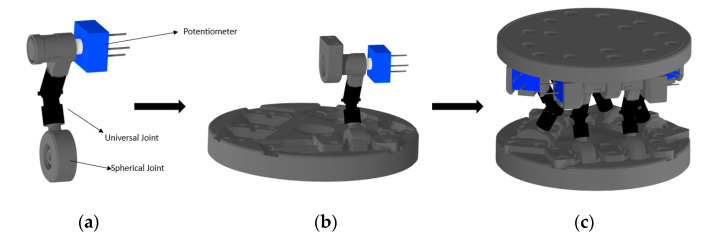
Modeling of the 6 DOF displacement sensor. (**a**) Configuration of link. (**b**) Attachment of link and plate. (**c**) assembled sensor.

The 6 DOF parallel mechanism can be implemented in various types by combining various joints differently. The most widely used mechanism is the SPS mechanism known as the Stewart platform. Additionally, there are many types such as the 3-3, 6-3, and 6-6 in accordance with the number of the joint vertex on the base platform and moving platform. The sensor proposed in this study was the 6-6 RUS parallel mechanism, as shown in Figure 5. The 3-3 and 6-3 parallel mechanisms are difficult to manufacture because they need to connect two links using one joint. Besides, there exist singularities that do not appear in the 6-6 parallel mechanisms [19]. Compared to linear displacement sensors such as LVDTs, rotational displacement sensors such as potentiometers and encoders are relatively smaller and cheaper. Thus we could make a compact and inexpensive system using rotational sensors.

An essential part of using the 6DOF parallel platform as a sensor is to avoid singularity. If a sensor is made based on the model discussed in Section 3.1, it is possible to prevent the singularity, and accurate sensing becomes possible. The sensor was manufactured in the form of the modified model discussed above to avoid singularity. Each leg of the sensor consisted of one revolute joint, one universal joint, and one spherical joint. It was possible to calculate the RUS platform’s end-effector position based on the angle of the revolute joint. Thus, a basic sensor that could measure the angle of the revolute joint was configured. In order to manufacture the sensor, it was necessary to reduce the tolerance of each joint. We used a universal joint (MAA-2.0, MIYOSHI KIKAI, Hirano, Japan) and a spherical joint (KGML W300, IGUS, Cologne-Porz, Germany) to reduce tolerances. The revolute joint had a structure in which the shaft rotates, therefore, we needed to catch the torsion caused by gravity or external force to make an accurate sensor. Through the installation of bearings and supports, a model that could minimize bending and tolerance was completed. In addition, a cylindrical shaft with a diameter of 2 mm was inserted between each joint. Figure 5a shows one link that went through this manufacturing process. The link could be attached to the plate in the direction of the model type discussed, as shown in Figure 5b. Figure 5c shows the assembled sensor with two plates and twelve links. 

In this study, potentiometers were used to measure the rotational displacement. The output signal was an analog signal, so the resolution could be significantly increased depending on bits of the ADC. We used potentiometers that had a 0~260 electrical rotational range. We converted it to a 10-bit ADC, so the resolution was 0.254°. In this case, the linear resolution was within 0.1 mm and 0.2 mm in the z-axis and x-axis, respectively. The diameter of the base platform and the moving platform was 38.934 mm, and the lengths of the two links were 14.795 mm and 14.8 mm, respectively. The upper and lower plates were both 60 mm in diameter, and the maximum height of the sensor was 41 mm when the universal joints were extended.

### 3.3. Experiment Procedure

**Figure 6:**
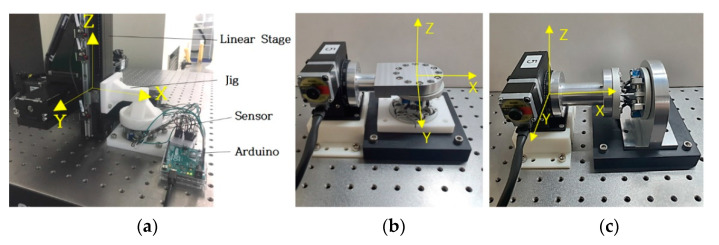
Experiment setting of the Displacement Sensor. (**a**) *x*, *y*, *z* axis experiment. (**b**) Roll, pitch axis experiment. (**c**) Yaw axis experiment.

Figure 6 shows the experimental setup to evaluate the developed displacement sensor. In this paper, the displacement measurements for the position [x y z] were tested with the base platform and the moving platform in parallel. The jig was made with a 3D printer to fix the sensor to the floor and the stage. The resolution of the 3-axis linear stage used was 0.06 mm. Moving the top plate along the z-axis by 0.5 mm at a time, we measured potentiometer values eight times so that the total movement was 4 mm. The X-axis and y-axis were measured in the same way. Since this model did not have a spring structure to sustain the initial position when no external force was applied, the experiment was conducted by setting an arbitrary position as the initial position. The experiment for measuring the 3-axis rotation angle was performed in the same way. In this experiment, a rotation stage and a 3D printed jig for each axis’s rotation were used. The resolution of this rotation stage was 0.025°. We observed a total of 8° by moving eight times by 1° in each axis. We could get an analog signal for the angle value of each revolute joint through the potentiometer. 

**Figure 7:**
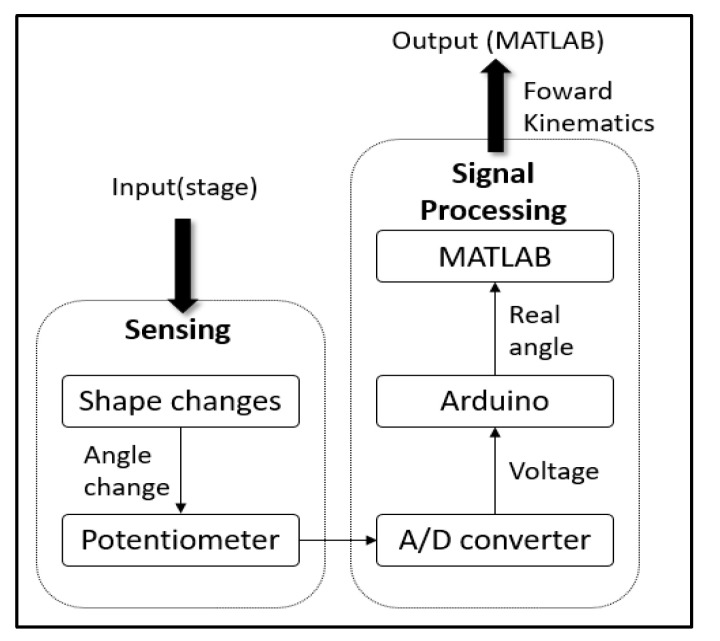
Schematic diagram of sensor verification experiment.

We converted it through an A/D converter and read the corresponding digital value through the Arduino. We brought it to MATLAB and substituted it into the forward kinematics (Equation (9)) to derive the final sensor value. Figure 7 shows the whole process of the experiment.

**Figure 8:**
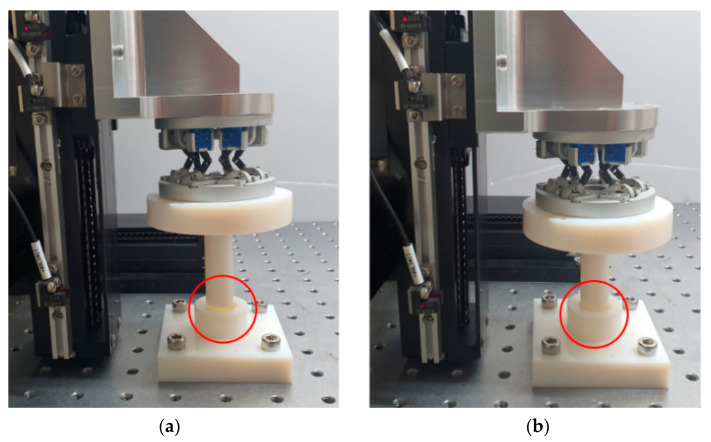
Experiment environment of the peg-in-hole system. (**a**) Experiment setting when misalignment with the hole is given (before peg-in-hole). (**b**) Successful peg-in-hole even with misalignment (after peg-in-hole).

There are various industrial applications based on 6-axis motion sensing. This paper proposed an industrial application plan by measuring misalignment through the sensor during simple peg-in-hole work. Figure 8 shows the setup of the peg-in-hole operation. A 3D printed hole and a cylinder-shaped bar suitable for the hole were used. The cylinder was attached to the developed sensor, and misalignment was forcibly applied using a linear stage to check whether the sensor could measure the misalignment. We gave a misalignment from −2 mm to 2 mm in increments of 1 mm in the *x* and *y*-axis by changing the *z*-axis two times (0 mm, 2 mm). Thus, we performed a total of 50 experiments to compare the difference with the actual misalignment. We verified whether the sensor could operate even when it is moved in various axes through this experiment.

### 3.4. Results and Discussion

**Figure 9:**
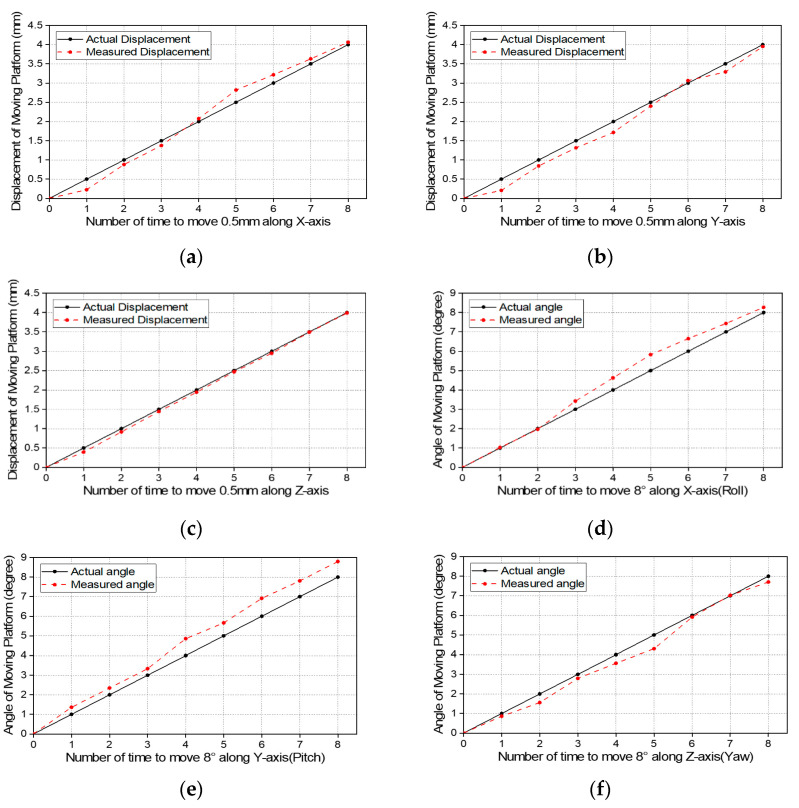
Result of the sensing experiment. (**a**–**c**) are x, y, z-axis sensing results. (**d**–**f**) are roll, pitch, yaw sensing results.

Figure 9 shows the results of the experiment. The black solid line means actual distance traveled by the stages which is defined as an input in Figure 7. The red dotted line indicates the value obtained by potentiometers which are defined as an output in Figure 7. Figure 9a–c show the result of the x-axis, y-axis, and z-axis, respectively. As a result, the error was measured within 0.1 mm in the z-axis experiment, and the measured error was within 0.3 mm in the x and y-axis experiments. Figure 9d–f show the experimental results in the roll, pitch, and yaw directions. In the yaw direction experiment, the error was within 0.5°, and the roll and pitch experiment showed an error within 0.8°. 

**Figure 10:**
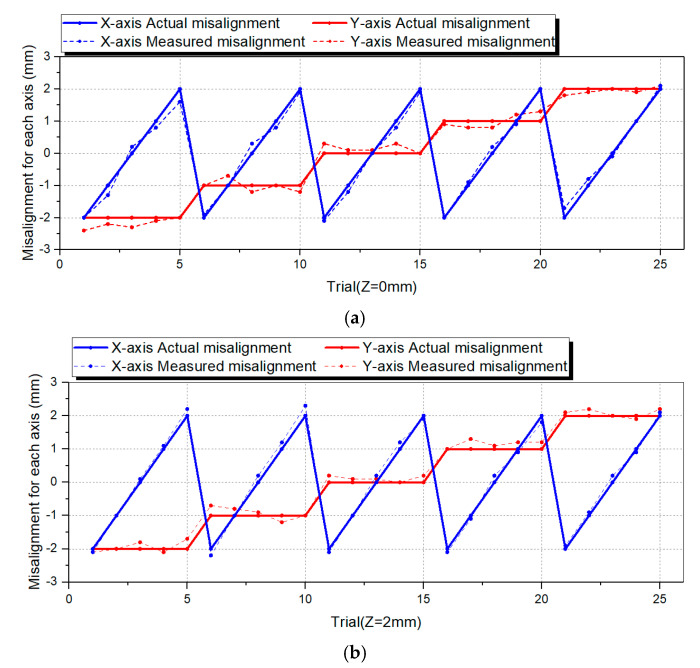
Result of the peg-in-hole misalignment sensing experiment for verifying sensing performance with axes combination. (**a**) Result of *x*, *y* axes misalignment experiment result when *z* axis misalignment is 0 mm. (**b**) Result of *x*, *y* axes misalignment experiment result when *z* axis misalignment is 2 mm.

As a result of the peg-in-hole experiment, we found that misalignment within 2 mm could be corrected through the RCC function of the manufactured sensor. Moreover, it was possible to estimate the length and direction of the misalignment through the six-axis displacement and angle sensing information the existing peg-in-hole system uses with a complex algorithm for correction in case of error. This device has an RCC function and position sensing function, so it is possible to measure the misalignment simply through the amount of change in the plate’s x and y-axis. The misalignment of the x and y axis was tested by changing each axis from (−2, −2) to (2, 2) mm in increments of 1 mm, and two different z-axis values were tested. For the analysis of the results, we calculated the misalignment of each of the *x* and *y*-axis when the *z*-axis was 0 mm and 2 mm and compared it with the actual value. Figure 10a,b shows the result when the *z*-axis is 0, 2 mm. The blue and red solid lines represent the real misalignment of the x and *y*-axis, and the blue and red dotted lines represent the measured x and *y*-axis misalignment. As a result, we found that even when the axes were combined, an error of within 0.4 mm was observed for each axis.

There are two main causes of error. First, tolerances generated during manufacturing parts and small gaps between parts in the assembly process accumulate and affect the result. As an example, the tolerance of the spherical bearing is measured at a maximum of 0.5 mm, so a 0.5 mm error can be made in the final measured value. Next, the noise and resolution of the potentiometers affect the results. Potentiometers have analog output, so they are vulnerable to noise generated by heat and vibration. In addition, the tolerance of the potentiometer due to the use of 10bit ADC is 0.254°, and the maximum tolerance of the device that can appear from 10bit ADC is 0.2 mm/0.6°. In addition, other errors may affect the results, but the main errors presented above can be reduced. 

The first error can be eliminated by applying pre-pressure to the spherical bearing. Applying pressure can reduce the range of motion of the spherical bearing. However, since the model produced in this study does not require a large range of motion for each joint, a reduction in the range of motion of the spherical bearing will not seriously affect the performance. The second error can be solved through a good filter and an improved experimental environment. When using a better ADC, it is possible to reduce the tolerance of the potentiometer, which can help improve performance. Due to the noise problem, there is currently no significant performance improvement at this time. In the future, we can expect performance improvement by removing the noise by using a filter and experimental environment improvement. Finally, the overall sensor calibration can reduce errors. 

**Figure 11:**
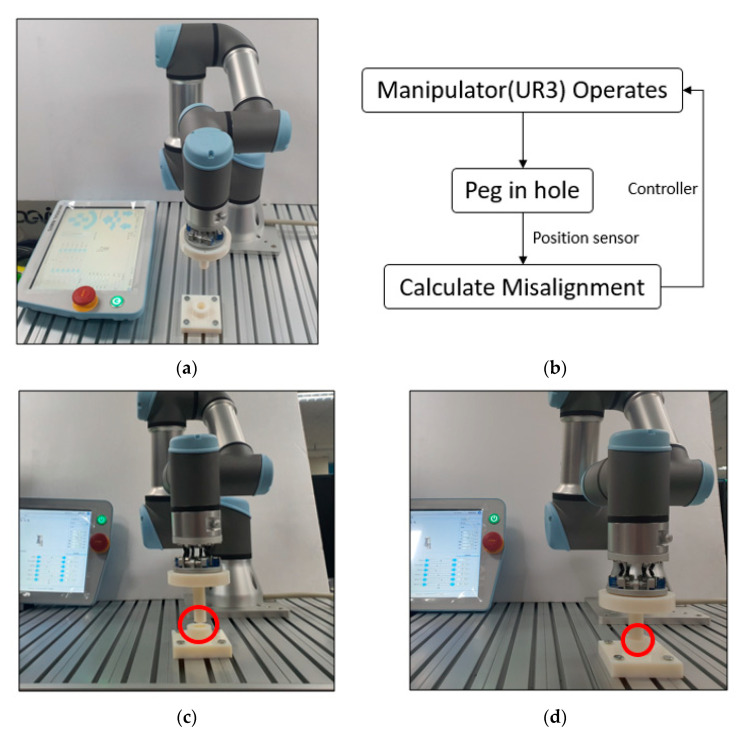
Peg-in-hole process with a UR3. (**a**) Sensor combined with a manipulator. (**b**) Peg-in-hole process diagram based on position control. (**c**,**d**) Verification of RCC function of the sensor through the peg-in-hole experiment.

**Figure 12:**
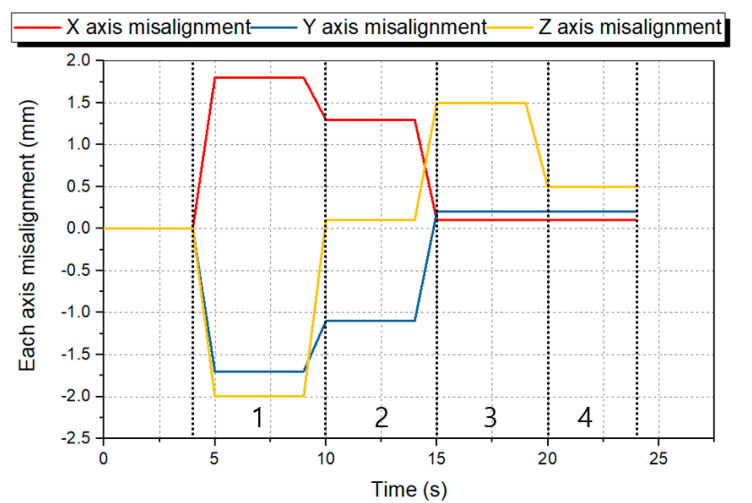
Result of peg-in-hole process experiment with random misalignment using a UR3.

We can check the operation of the sensor by attaching the sensor to the actual manipulator. In this study, by performing a peg-in-hole experiment using UR3 (Universal Robots, Denmark), we verified that the sensor can be attached to the manipulator to play the role of the RCC. Figure 11 shows the experiment using UR3 and the process of the peg-in-hole experiment. Based on this experiment method, we conducted a simple feedback control when random misalignment occurs using a teaching pendant. Figure 12 shows this result. Zone 1 in Figure 12 shows the measured *x*, *y*, *z* axes misalignment result value when performing the peg-in-hole experiment after randomly placing the hole position. After lifting the peg from the hole in the *z*-axis direction, the result of misalignment of the *x*, *y*, and *z* axes appears in zone 2. We performed the second peg-in-hole experiment by correcting the *X* and *Y* axes based on the result of zone 1, and zone 3 shows the result. The result of misalignment of the *x*, *y*, and *z* axes after lifting the peg in the *z*-axis direction appears in zone 4. Through this result, we showed that it is possible to find misalignment through displacement sensing of the RCC and the possibilities to correct it in the next attempt.

## 4. Conclusions

We introduced a displacement sensor using the 6 DOF parallel mechanism so that it can measure the displacement of the end-effector. First, we analyzed the kinematics of the 6 DOF RUS parallel mechanism and showed that we can obtain the position and orientation of the moving platform from six angular displacement sensors. We also designed a sensor whereby singularities do not occur within the operating range through geometric structure transformation. Next, we manufactured an inexpensive and compact displacement sensor by using potentiometers and evaluated its performance through precision stages. 

As a result, an error occurred due to a 10-bit ADC. In some cases, more significant errors occurred. Through the search for the reasonable error cause, future research will be able to make a more precise sensor that reduces the errors. Through the peg-in-hole simulation, it was found that the instrument can correct misalignment of up to 3 mm. Besides, it showed the possibility of performing position sensing during calibration and showed that the manufactured instrument could be used for real-time control and prevention of damage to assembly robots in an industrial environment. Additionally, it is possible to manufacture a machine that can withstand stiffness in an actual industrial environment by adding a spring structure in the machine, and it is expected that 6-axis force/torque sensing can be performed in the future through the analysis of the spring. In addition, by performing peg-in-hole control using a manipulator with 6 axes F/T displacement sensor, it is expected to increase the effectiveness by simplifying the method for overcoming the misalignment of various processes. 

## Figures and Tables

**Table 1:** Results of the Forward Kinematics Solution.

Trial	Inverse Kinematics					
	*x*	*y*	*z*	*ψ*	*θ*	*φ*
1	5.30	2.40	−26.10	183.40	4.30	2.70
2	−3.72	4.57	−24.69	185.60	6.40	3.50
3	1.57	6.42	−22.00	175.00	−3.52	4.75
4	4.26	3.56	−25.00	178.30	3.31	4.20
5	−3.50	−3.50	−24.00	177.00	3.00	4.00
**Trial**	**Forward Kinematics**					
	***x***	***y***	***z***	***ψ***	***θ***	***φ***
1	5.30	2.40	−26.10	183.40	4.30	2.70
2	−3.72	4.57	−24.69	185.60	6.40	3.50
3	1.57	6.42	−22.00	175.00	−3.52	4.75
4	4.26	3.56	−25.00	178.30	3.31	4.20
5	−3.50	−3.50	−24.00	177.00	3.00	4.00

**Table 2:** Results of the Forward Kinematics of the Prototype and Modified Model.

Trial	Initial value of Numerical Solution					
	*x*	*y*	*z*	*ψ*	*θ*	*φ*
1	0.00	0.00	−25.00	180.00	0.00	0.00
2	0.00	0.00	−25.00	180.00	0.00	1.00
3	0.00	0.00	−25.00	180.00	0.00	3.00
4	0.00	0.00	−25.00	180.00	0.00	7.00
5	0.00	0.00	−25.00	180.00	0.00	10.00
**Trial**	**Prototype Model**					
	***x***	***y***	***z***	***ψ***	***θ***	***φ***
1	0.00	0.00	−25.0000	180.00	0.00	0.0000
2	0.00	0.00	−24.9957	180.00	0.00	0.8168
3	0.00	0.00	−24.9862	180.00	0.00	1.4556
4	0.00	0.00	−24.9762	180.00	0.00	1.9120
5	0.00	0.00	−24.9716	180.00	0.00	2.0898
**Trial**	**Modified Model**					
	***x***	***y***	***z***	***ψ***	***θ***	***φ***
1	0.00	0.00	−25.00	180.00	0.00	0.00
2	0.00	0.00	−25.00	180.00	0.00	0.00
3	0.00	0.00	−25.00	180.00	0.00	0.00
4	0.00	0.00	−25.00	180.00	0.00	0.00
5	0.00	0.00	−25.00	180.00	0.00	0.00

## Data Availability

Not applicable.
